# Role of ERG3 mutation and expression in azole resistant Candida albicans isolated from vulvovaginal candidiasis patients

**DOI:** 10.12669/pjms.41.3.9325

**Published:** 2025-03

**Authors:** Ronaq Zaman, Ihsan Ullah, Ambreen Arif

**Affiliations:** 1 Ronaq Zaman, MBBS, MPhil, PhD Scholar Department of Microbiology, Khyber Medical University, Peshawar, Pakistan; 2 Ihsan Ullah, MBBS, DFM, PGD EBM & HPE, PhD Associate Professor, Institute of Pathology and Diagnostic Medicine, Khyber Medical University, Peshawar, Pakistan; 3 Ambreen Arif, MPhil, PhD Scholar Department of Microbiology, Khyber Medical University, Peshawar, Pakistan

**Keywords:** Azole resistance, Candida albicans, ERG3 gene, Expression, Mutations

## Abstract

**Objective:**

The study aimed to investigate mutations and mRNA expression of the *ERG3* gene in resistant *Candida albicans* isolates.

**Methods:**

This cross-sectional study was conducted from October 2018 to June 2019. High vaginal swab samples were collected from Hayatabad Medical Complex and transported to Khyber Medical University. Samples were inoculated on different media and identified by 20C AUX strips. Antifungal susceptibility was determined using the disc diffusion and broth microdilution methods. The *ERG3 gene* was amplified and sequenced to find amino acid polymorphisms. Real-time PCR was performed to study level of *ERG3* expression.

**Results:**

A total of seventy-three (n=73) C*andida albicans* out of 369 samples were isolated. Among the isolates 49.3%, 54.8%, 53.4%, 47.9%, 30.1% were resistant to fluconazole, Clotrimazole, Miconazole, Voriconazole and Itraconazole, respectively. Sanger sequencing of *ERG3* gene of isolates revealed six synonymous mutations. Expression level of mRNA of *ERG3* gene in azoles sensitive stains (3.72±2.22) was higher than those in the resistant *Candida albican* strains (1.74±0.96).

**Conclusion:**

This study revealed synonymous mutations and low expression of *ERG3* gene in azole-resistant *C. albican*.

## INTRODUCTION

Among yeasts the *Candida* infections are the most frequent. The *Candida* is involved in local as well as disseminated infections.^1^ Vulvovaginal candidiasis (VVC) is the most frequent female genital tract infection. The common specie isolated in VVC is *Candida albicans*. It is reported that at least one episode of candidiasis occurs in 75% of women. Among these 8% have chronic recurrent VVC (RVVC) episodes.^2^ Use of antibiotics, contraceptives, personal hygiene, diabetes mellitus and immunological defects have been recognized as risk factors.^3^

Azole antifungal drugs are usually prescribed for the treatment of *Candida* vaginitis. Commonly prescribed azoles drugs are Fluconazole, Clotrimazole and Miconazole. Widespread use of azoles drugs has led to resistant *Candida* strains.^4^ Different mechanisms of resistance can occur simultaneously in azoles resistant isolates. Azoles interferes with ergosterol synthesis. Lanosterol 14α-demethylase enzyme is inhibited, which is coded by *ERG11* gene. Point mutations in the *ERG11* gene is the common resistance mechanism in *Candida* species against azoles. More than 140 missense mutations in *ERG11* gene in *Candida albicans* have been documented by several researchers.^5^ Other resistance mechanisms are efflux pumps overexpression, *ERG11* and *ERG3* gene mutations. In *Candida albicans*, *ERG3* gene encodes Δ desaturases. Which is responsible for conversion of nontoxic metabolites accumulated in azole inhibition to toxic sterols.^5^,^6^

Inactivation of desaturases enzyme due to *ERG3* mutations and low expression leads to reduced effects of azoles on yeast cells. *ERG3* gene mutations are enough to induce azole resistance in *Candida* species.^6^ Point mutations that do not change protein sequence are called synonymous or silent mutations. Nonsynonymous mutations alter the protein sequences. Synonymous mutations were presumed to be neutral, but most of them are deleterious. Non-synonymous and synonymous mutations both alter gene expression levels.^7^

A better understanding of the resistance mechanisms of azole resistant Candida species will facilitate the design of more effective strategies to prevent and overcome resistance to antifungal agents. However amino acid substitutions and active efflux are the most common azole resistance mechanism in *C. albicans*. Enzymes of the ergosterol biosynthesis pathway, such as *ERG3* are poorly studied.^8^ The current study aimed to investigate expression and mutations of the *ERG3* gene in azole resistant *Candida albicans*.

## METHOD

This cross-sectional study was conducted from October 2018 to June 2019 from patients in Hayatabad Medical Complex, Peshawar, Pakistan. Sampling technique was nonprobability convenient. Using aseptic techniques two high vaginal swabs (HVS) were taken from each patient.^9^ A total of 369 HVS were collected from vulvovaginitis patients of child bearing females. High vaginal swab samples were transported to Khyber Medical University. Samples were inoculated on Sabauroud’s dextrose agar. *C. albicans* form creamy white colonies on Sabaurouds agar.^9^ *C. albicans* were identified with Candida chrom agar and 20 C AUX strips.^10^ Antifungal susceptibility was evaluated by disc diffusion and broth microdilution methods according to Clinical and Laboratory Standard Institute Standards (CLSI). The susceptibilities of yeast isolates to fluconazole (25µg), clotrimazole (10µg), miconazole (50µg), voriconazole (1µg) and itraconazole (10µg) were determined.^11^

### Ethical approval

(No; Dir/KMU-EB/IM/00608; Dated: 11-09-2018) was obtained from the Institutional ethical committee, Khyber Medical University, Peshawar. From all patients a written informed consent was obtained.

Extraction of DNA from *C. albicans* isolates was performed by Cetyltrimethylammonium bromide (CTAB) method. The method was used with modifications. A loop full of fresh *C. albicans* colonies were transferred to 100µl of distilled water in ependrope tube. It was centrifuged at 12000rpm for five minutes. Supernatant was discarded and 200µl of TE buffer and 50µl of lysozyme ((10mg/ml) were added to the pellet. It was kept overnight in shaking water bath at 37^0^C. Then 70µl of 10% SDS (sodium dodecyl sulfate) and 5µl proteinase k (20mg/ml) were added and incubated for 10 minutes at 65 ^0^C. Then 100µl of 5M NaCl and 100l µl CTAB/NaCl buffer was added and incubated for 10 minutes at 65^0^C. Then 750ml of chloroform/isoamyl alcohol was added in 24:1 ratio. Centrifugation for 10 minutes at 10,000g was done. Then the supernatant was transferred to other ependrope tube and 450µl of 100% isopropanol was added to the supernatant. The samples were centrifuged at 10,000g at 15minutes. Then the pellet was washed with 70% ethanol and centrifuged at 10,000g for 10minutes. Ethanol was removed and 50µl of TE buffer was added to the pellet and kept at 40^0^C overnight to rehydrate DNA. DNA was then stored at -20^0^C.^12^,^13^

The *ERG3* gens was amplified in twenty-five resistant and two sensitive *C. albican* isolates the primers used were *ERG3-F* 5’ATGGATATCGTACTAGAA3’ and *ERG3-R* 5’TCATTGTTCAACATATTC3’.^14^ Amplified products were sequenced to identify mutations. Bio edit and Clustal W were used for sequence data analysis.^15^ Forty-three resistant and seven sensitive isolates were selected for reverse transcriptase real time -PCR study. Expression levels of *ERG3* gene were determined. Isolates were grown on Sabaurouds agar. RNA was extracted by trizol method.^16^ The total RNA was quantified with a spectrophotometer. Reverse transcription was performed using the cDNA kit (Cat # G234, One Script® ABMGood, Canada) The quantitative PCRs of *ERG3* were performed in duplicates by 7500 Real-Time PCR.^17^,^18^ The primer used were *ERG3 F* 5’ TCC AGT TGA TGG GTT CTT CC 3’ and *ERG3R* 5’ GGA CAG TGT GAC AAG CGG TA 3’ used were. The ACT1 gene was used as a reference gene.^19^

### Statistical analysis:

For data analysis SPSS version 20 was used. Percentages were calculated for categorical variables. Results were stated as means ± standard deviations.

## RESULTS

Out of total 369 patients, *Candida albicans* were isolated from 19.78% (n=73). Age range of the patient was 18-46 years. Age distribution was as follows; 17.8%(n=13) of patients were of 18-23 years, 41.09%(n=30) of patients were of 24-29 years, 31.5%(n=23) of the patients were in the range of 30-35 years, 6.8%(n=5) of patients were in range of 36-41 years, 2.7%(n=2) of the patients were in range of 42-46 years.

**Fig.1:**
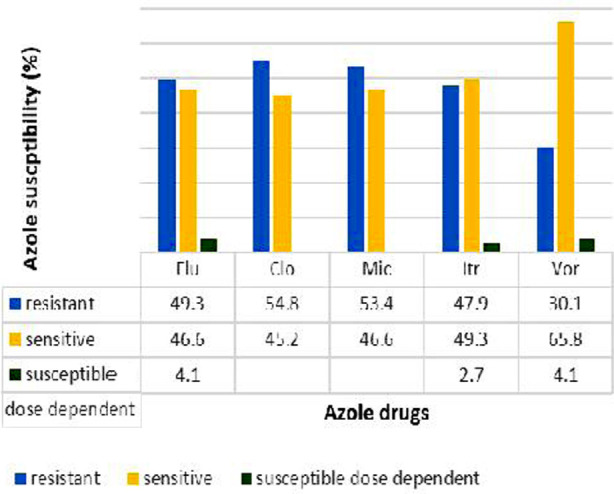
Azole susceptibility of Candida albicans.

**Fig.2:**
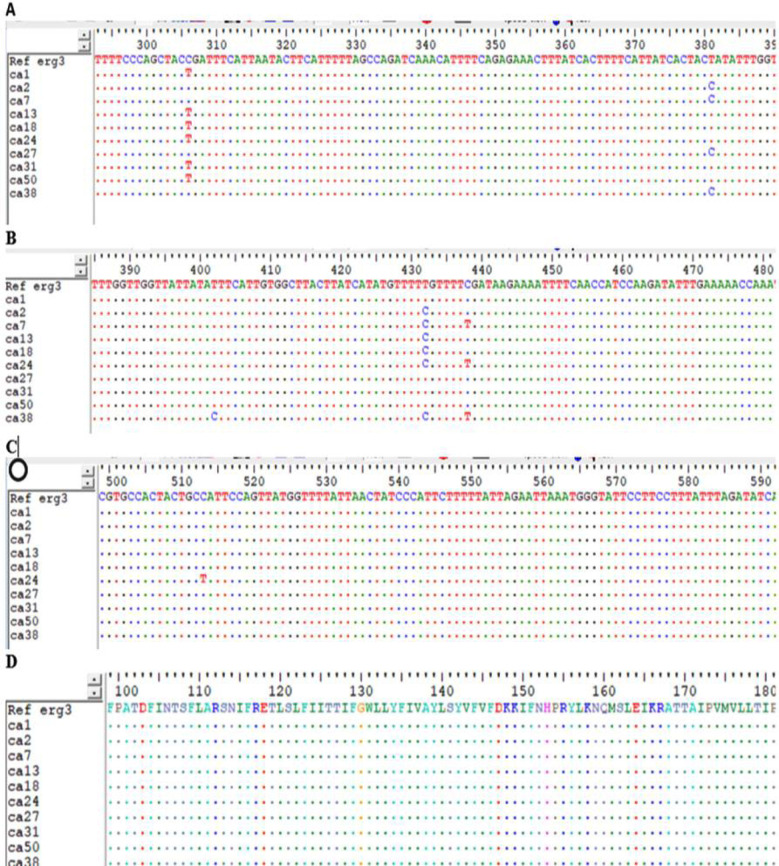
Alignment of sequences of erg3 gene of Candida albicans isolates with reference strain (SC5314) ERG3gene A, B, C shows the nucleotide substitution. D showing no change in the amino acid sequence.

Among the isolates 49.3%(n=36) were Fluconazole resistant, 54.8%(n=40) were resistant to Clotrimazole, 53.4%(n=39) were resistant to Miconazole, 47.9%(n=35) were resistant to Voriconazole and (30.1%(n=22) were resistant to Itraconazole (Fig.1). Pan-azole resistant isolates were 15%(n=11). Cross resistance to the azoles drugs was revealed among the isolates. Sequence results were analyzed by alignment with *ERG3* sequence of reference strain, *Candida albicans* SC5314. C306T (T102T), T381C (T127T), 402CT (Y134Y), T432C (F144F), C438T (F146F), C513T (A 171A) were the synonymous mutations detected (Fig.2). C513T is a novel synonymous mutation. One of the azole sensitive isolate (CA 50) showed only one, C306T mutation as shown in Table-I. There was no missense mutation detected in the sequenced isolates. The sequences of the *ERG3* gene of *Candida albicans* isolates containing synonymous mutations were submitted to gene bank (accession # ON254177-ON254195).

**Table-I:** Antifungal susceptibility, Fold increase in expression and point mutations in ERG3 in Candida albican isolates.

Isolates	MIC (|jg/ml)					Fold increase in expression	Mutations in erg3 gene

	FLC	CLO	MIC	VOR	ITR		
Ca1	64	0.5	4	8	0.125	0.36	C306T
Ca2	64	0.5	2	4	0.125	1.5	-
Ca3	64	1	16	4	0.125	1.1	T381C.T432C
Ca4	2	0.5	8	0.5	0.125	3.6	-
Ca5	64	2	4	4	0.125	3.1	T381C.T432C
Ca6	1	0.5	2	0.5	0.125	2.0	-
Ca7	1	0.5	16	0.5	0.125	3.2	T381C, T432C, T438C
Ca8	64	2	16	1	0.5	1.8	-
Ca9	2	0.5	2	0.5	0.125	1.5	C306T
C10	16	0.5	1	0.5	0.125	1.8	-
Ca11	64	1	4	0.5	0.125	1.5	-
Ca12	64	2	16	0.5	0.125	2.1	-
Ca13	2	4	16	4	1	1.5	-
Ca14	64	1	8	1	0.125	0.61	-
Ca15	64	1	16	1	1	6.0	-
Ca16	64	2	16	1	0.125	5.0	-
Ca17	64	2	32	2	0.125	2.8	T381C, T432C, T438C
Ca18	64	1	16	1	0.125	5.3	-
Ca19	2	1	60	1	0.125	1.9	-
Ca20	2	1	1	0.5	0.125	1.3	-
Ca21	64	2	32	1	0.125	3.6	-
Ca22	64	1	16	1	1	0.49	-
Ca23	2	0.5	16	1	0.5	2.3	-
C24	64	2	16	4	2	1.6	T381 C,T402C,T432C,C438T
Ca25	64	1	16	1	1	3.1	-
Ca26	64	1	16	4	1	1.8	C306T,T432C
Ca27	64	0.5	1	0.5	1	7.0	C306T,T432C,C513T
Ca28	64	2	16	0.5	1	1.1	C306T,T432C, C513T
Ca29	64	1	16	1	0.125	1.8	C306T,T432C
Ca30	64	1	32	4	1	1.2	C306T,T432C,C438T,C513T
Ca31	64	0.5	8	1	0.125	1.2	T432C, C513T
Ca32	0.5	1	0.5	0.125	0.125	1.4	C306T
Ca33	64	4	16	0.5	0.125	1.8	-
Ca34	4	2	16	0.5	0.125	1.0	-
Ca35	64	0.5	4	1	0.125	1.3	-
Ca36	4	0.5	2	0.5	0.125	1.8	-
Ca37	64	0.5	16	0.5	0.125	2.4	-
Ca38	64	0.5	16	0.5	0.12(S)	1.0	-
Ca39	64	4	1	4	0.12(S)	1.8	-
Ca40	64	4	16	2	0.12(S)	1.6	-
Ca41	64	2	1	1	0.12(S)	1.2	-
Ca42	R	2	16	0.5	1	0.27	-
Ca43	64	1	16	0.5	1	1.1	-
Ca44	4	0.5	4	0.5	0.125	2.1	-
Ca45	1	0.5	2	0.5	0.125	1.7	-
Ca46	4	0.5	2	0.5	0.125	2.1	-
Ca47	2	0.5	4	0.5	0.125	3.7	-
Ca48	1	0.5	2	0.5	0.125	1.6	-
Ca49	4	0.5	1	05	0.125	3.5	-
Ca50	2	0.5	1	0.5	0.125	1.4	C306T
FLC fluconazole CLO clotrimazole MIC miconazole ITR itraconazole VRC voriconazole							

Relative gene expression levels were calculated by 2−ΔΔCt method. Over expression was considered if the expression levels of target genes of the resistant isolates were more than that of *C. albicans* ATCC 90028. The expression levels of *ERG3* gene in the isolates are given in Table-I. The results showed that m RNA levels of *ERG3* gene in azoles susceptible isolates (3.72±2.22) were higher than those in azoles resistant *Candida albicans* isolates (1.74±0.96).

## DISCUSSION

In this study *C. albicans* was found to be 19.78%. The prevalence rate is consistent with other studies done previously in Pakistan, which reported isolation of *Candida albicans* from 12% and 10% of patients.^20^,^21^ Vulvovaginal candidiasis is common in reproductive age women. In reproductive age hormones are on its peak. Estrogen cause deposition of glycogen in vagina which enhances growth of *candida*. The current study revealed high rates of infection in age range of 24-35years as 41.09%(n=30) of patients were of 24-29 years, 31.5%(n=23) of the patients were in the range of 30-35 years. Similar findings were reported by Disha et al.^22^

The widespread use of azoles has resulted in increased resistance in azoles. Among azoles Clotrimazole, Fluconazole and Miconazole are frequently used. The results of this study revealed increased resistance in *Candida albicans* to Clotrimazole (54.8%), Fluconazole (49.3%) and Miconazole (53.4%) while low resistance was seen to less frequently used drugs like Itraconazole (30.1%). The results of susceptibility pattern regarding Itraconazole are consistent with a study done by Manza et al.^23^ The results revealed 88.33% of isolates sensitivity to Itraconazole while Fluconazole and Clotrimazole resistance among isolates found was lower than the current study. In another study conducted in Lebanon revealed high susceptibility of *Candida albicans* isolates to Voriconazole (97.5%), Itraconazole (87.5%) and Fluconazole (90%). The resistance against Clotrimazole, Miconazole and Fluconazole is more than other population due to excessive and irrational usage of antifungal drugs.^24^

In the current study Sanger sequence results of *ERG3* gene revealed synonymous mutations while no missense mutation was found. In past decade researchers recognized the synonymous mutations to be non-neutral. Synonymous mutations were found to be 75.9% deleterious in study done by Shen et al. They also investigated the mechanisms by which silent mutations affect the fitness. It was revealed that synonymous mutations vary the level of gene-expression.^7^ Thus, whether silent mutations are involved in azoles resistance remains to be explored.

Our results are similar to the study conducted by Zhang et al, which revealed the synonymous mutations.^14^ Synonymous mutations were presumed to be neutral and were generally ignored in studies. Feng et al reported 2 missense and one nonsense mutation in *ERG3*.^25^ The results are not in agreement with this study other mechanisms of resistance may be involved in resistance to azoles in *C. albicans* isolates. The results of Morio and Spettel et al are also in contrast to our study as they also reported missense mutations.^8^,^26^ The reason may be that the strains are isolated from vulvovaginal patients while other studies have recovered the strains from different sites of the body like blood, mouth and respiratory tract.

The results revealed that the *ERG3* gene expression levels in azoles susceptible isolates were higher than those in azoles resistant *Candida albicans* isolates. The mechanism might be that *ERG3* overexpression increases the production of sterol Δ 5, 6 desaturases. Which converts the nontoxic intermediates to toxic steroids. As a result, death of fungi is accelerated due to damage to the fungi cell membrane. The results are in accordance with the study conducted in by Feng etal.^25^ In a study by Akins et al also indicated that high expression of *ERG3* leads to increased susceptibility to drugs.^27^ High expression of *ERG3* gene were speculated to rise the susceptibility azoles in *C. albicans* in this study. Other genes of ergosterol pathways and efflux pump genes needs to be investigated, which could be the reason for the azole resistance in *C. albicans*.

### Limitations:

This study was conducted in one hospital of Peshawar. it might increase our knowledge about the resistance profile and it’s causes if large number of samples are selected from different hospitals.

## CONCLUSION

This study results revealed silent mutations in *ERG3* gene. Azole resistant isolates of *C. albicans* showed low expression of *ERG3*.

### Recommendations:

Mechanisms of resistance like other genes of ergosterol pathways and efflux pump genes needs to be explored, which may be the reason for the high resistance to azoles in *c. albicans*.

### Authors Contribution

**RZ:** Literature search, data collection, laboratory work, manuscript writing

and responsible and accountable for the accuracy or integrity of the work.

**IU:** Concept & project design, review, editing and final approval of manuscript.

**AA:** Did data analysis and interpretation. Critical review.

All authors have approved the final version and are accountable for the integrity of the study.
